# Immediate 3-dimensional ridge augmentation after extraction of periodontally hopeless tooth using chinblock graft

**DOI:** 10.4317/jced.52366

**Published:** 2015-12-01

**Authors:** Ankit Desai, Raison Thomas, Tarunkumar A. Baron, Rucha Shah, Dhoom-Singh Mehta

**Affiliations:** 1 MDS, Senior Lecturer, Department of Periodontics & Oral Implantology, Ahmedabad Dental College & Hospital, Bhadaj - Ranchodpura Road, Ahmedabad- 382115 Gujarat, India; 2 MDS, Professor, Department of Periodontics, Bapuji Dental College and Hospital, Davangere-577004, Karnataka, India; 3 MDS, Lecturer, Department of Periodontics, Bapuji Dental College and Hospital, Davangere-577004, Karnataka, India; 4 MDS, Professor and Head, Department of Periodontics, Bapuji Dental College and Hospital, Davangere-577004, Karnataka, India

## Abstract

**Background:**

The aim of the present study was to evaluate clinically and radiographically, the efficacy of immediate ridge augmentation to reconstruct the vertical and horizontal dimensions at extraction sites of periodontally hopeless tooth using an autogenous chin block graft.

**Material and Methods:**

A total of 11 patients (7 male & 4 female) with localized advanced bone loss around single rooted teeth having hopeless prognosis and indicated for extraction were selected for the study. The teeth were atraumatically extracted and deficient sites were augmented using autogenous chin block graft. Parameters like clinically soft tissue height - width and also radiographic ridge height -width were measured before and 6 months after augmentation. Obtained results were tabulated and analysed statistically.

**Results:**

After 6 months of immediate ridge augmentation, the mean gain in radiographic vertical height and horizontal width was 7.64 + 1.47 mm (*P* = 0.005) and 5.28 + 0.46 mm (*P* = 0.007) respectively which was found to be statistically significant (*P* < 0.05). Mean change of width gain of 0.40mm and height loss of 0.40mm of soft tissue parameters, from the baseline till completion of the study at 6 months was observed.

**Conclusions:**

The present study showed predictable immediate ridge augmentation with autogenous chin block graft at periodontally compromised extraction site. It can provide adequate hard and soft tissue foundation for perfect 3-Dimensional prosthetic positioning of implant in severely deficient ridges.

** Key words:**Immediate ridge augmentation, periondontally hopeless tooth, autogenous chin graft, dental implant.

## Introduction

Dental implantology is one of the accepted and predictable treatment approaches for restoring lost teeth. Consequently, as the practice of implant rehabilitation has developed and matured, both patients and the reconstructive team have reframed their treatment expectations (1). Implant rehabilitation is no more simply a vehicle to restore lost masticatory and phonetic function; but its horizons extended to achieve ideal treatment outcomes in terms of structural and esthetic rehabilitation of edentulous spaces.

When replacing missing teeth, available options include removable partial denture [RPD], conventional fixed bridge [FPD] or implant supported prosthesis. As known, RPD is a poor treatment option to restore esthetic and function. FPD has long been a treatment option with survival rates of 89.1% and 81.1%, respectively, at 5- & 10- years (2). But for single tooth replacement by FPD requires crown preparation of the adjacent teeth which increases incidence of caries, sensitivity, mobility and periodontal breakdown of the abutment teeth (2). Implant supported prosthesis are emerging as a newer treatment modality with the 5- and 10-year survival rates of implant-supported single crowns estimated to be 96.3% and 89.8%, respectively (3). Implant treatments eliminates necessity of the abutment teeth. Thus over the past few decades implant dentistry has grown in scope due to the demonstrated success and predictability such that the clinicians around the world consider it to be a form of a standard care (4).

Post-extraction healing response of an alveolar socket at 6 months shows as much as 40% of ridge height loss and 60% of ridge width loss followed by soft tissue shrinkage (5). The resorptive pattern gets worse with pre-existing conditions like periodontal, periapical infections or vertical fracture with infected sinus tract. As a result, often in clinical practice, the deficiency of bone volume is the primary reason for avoiding implant treatment (6). For prosthetically determined implant placement, final prosthesis type & design dictates the number, size and the ideal implant position (7). Such situations can be managed by bone augmentation to re-establish the ridge volume (8).

As dimension changes in normal sockets are significantly different in augmented vs unaugmented sockets (9), it is even more important in case of infected sockets with advanced bone loss [such as that of periodontally hopeless teeth] to augment immediately after extraction to avoid severe post extraction bone loss. Kfir *et al.* found that immediate ridge augmentation [IRA] using titanium membrane after extraction of the infected tooth was a successful and safe procedure. All patients of the study achieved sufficient bone augmentation and 8 patients received implants without any additional guided bone regeneration (10).

Despite recent advances in bone grafts & bone-substitute technology, intra membranous autogenous osseous transplants are regarded as the gold standard for reconstruction of the deficient alveolar ridge (11). If the amount of bone necessary for the augmentation is modest, grafts can be easily obtained from mandibular symphysis as it has an excellent risk-benefit ratio (11,12). However, during chin graft harvest, sensory dysfunction may occur due to traumatic edema to the epineurium or direct damage to mandibular incisive canal [MIC] (13,14). Radiographic assessment based new safety guidelines have been proposed by Pommer *et al.* to harvest chin graft aiming to reduce the neurosensory disturbances to mandibular anterior teeth (14). To the best of our knowledge, there are no studies so far evaluating the efficacy of new safety guidelines (14) to harvest chin graft.

Several reconstruction procedures using chin graft have been proposed to increase alveolar ridge volume (15,16).However, there is a paucity of literature regarding simultaneous reconstruction of ridge volume using the autogenous grafts immediately after extraction of a periodontally hopeless tooth. Hence, this study was performed with an aim to evaluate the success of 3-dimensional ridge augmentation over a period of 6 months, clinically and radiographically, with the use of an autogenous chin block graft immediately after extraction of periodontally hopeless tooth.

## Material and Methods

The patients for this study were selected from the Institutional Outpatient Department. Each patient was given a detailed verbal & written description of the risk & benefits of the proposed treatment in their own language and a signed informed consent was obtained from them before commencement of the study. Ethical approval for the study was obtained from the Institutional Ethical Committee.

**Figure 1:**
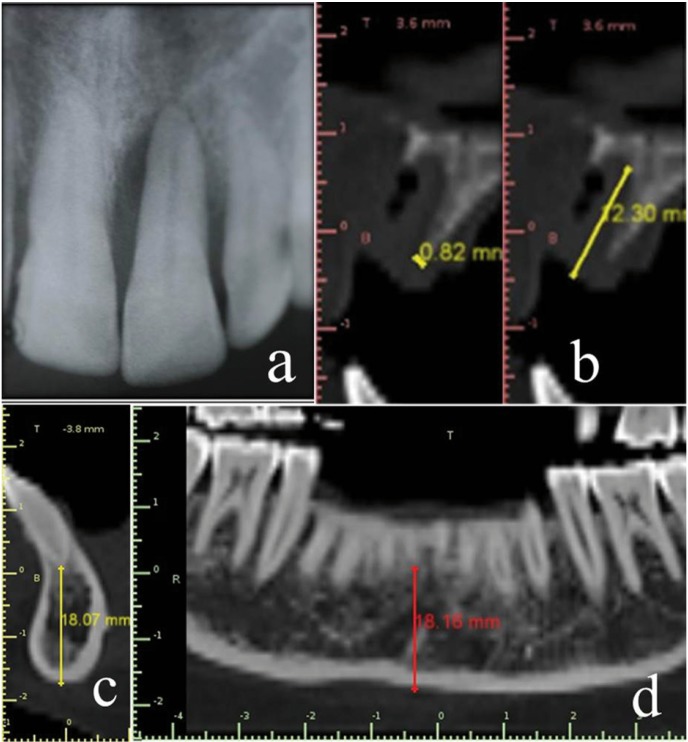
Pre-operative radiographic view of the recipient and donor site a) Periodontally hopeless tooth. b) Height and width of the deficient ridge immediately after extraction. c,d) Determination of safety outline for chin graft harvest.

A total of 11 patients [4 females and 7 males] in the age group of 18- 45 years [mean, 37.7 + 4.9 years] were selected for the study. Patients with a single periodontally hopeless (17) maxillary or mandibular single rooted tooth (grade-III mobility and/or advanced bone loss on radiographs) indicated for extraction with adjacent recession-free healthy site were considered ideal for study (Fig. 1a). Subjects with compromised medical history, such as diabetes & hypertension, and smokers were excluded.

Detailed medical and dental histories were recorded. Laboratory investigations including complete haemogram, bleeding and clotting time, glycated haemoglobin [HbA1c] assay, rapid ELISA and HBsAg were performed to evaluate the general fitness of the patient for the surgery. Diagnostic casts were prepared. All the patients received an initial therapy of oral hygiene instructions, scaling and root planing.

**Figure 2:**
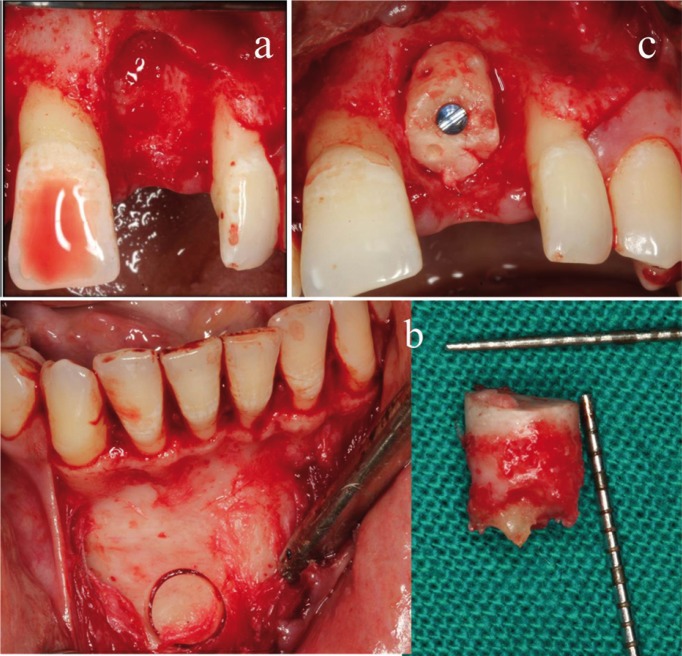
Clinical view of a) Recipient site after thorough curettage. b) Chin bone core harvest by trephine bur. c) Graft stabilization at the recipient site.

**Figure 3:**
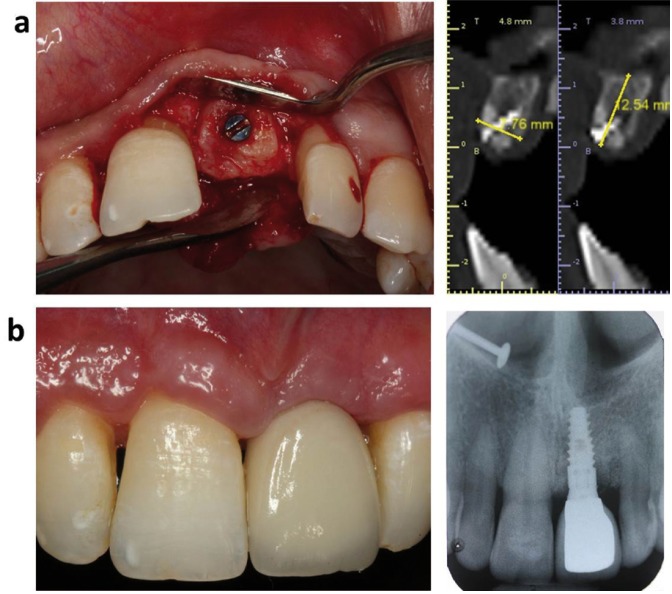
a) Post-augmentation clinical and radiographic view at 6 months recall. b) Clinical and radiographic view after Implant Restoration.

Recorded clinical parameters, at baseline and 6 months post-augmentation, include clinical photographs, soft tissue dimensions [height and width] using UNC-15 periodontal probe [Hu-Friedy, USA] and a stent. Radiographs included intra-oral periapical, panoramic, lateral cephalometric radiographs and a computed tomographic scan [CT Scan]. The linear measurements for ridge height and width were made on a CT scan from a fixed reference point [Cemento-enamal junction (CEJ) of the adjacent mesial tooth] (Figs. 1b-3a) by using Blue Sky Bio® software [Version 3.19.24, Anne solutions, Germany].

-Surgical method: All the patients were given premedication in form of one tablet of Augmentin 625 mg [500 mg amoxycillin & potassium clavulanate equivalent to 125 mg clavulanic acid] along with Tab. Ketorolac DT 10 mg [Ketorolac] one hour before the procedure. One-time steroid therapy with IV Dexamethasone phosphate 8mg/2ml inj. was given to control the postoperative swelling and edema. Pre-surgical scrubbing was done on the extra oral surface with 5 % Povidine iodine (18). Likewise, the patient was asked to perform pre-surgical mouth rinse with 0.2% chlorhexidine gluconate for 1 min, to lower the bacterial count of the oral cavity (19,20). All the surgeries were conducted under local anaesthesia using lidocaine 2% with adrenaline [1:100,000].

-On the recipient site: Crevicular incisions were placed around the tooth indicated for extraction with no. 15c B.P blade in such a way that adjacent interdental papilla or the tooth was not damaged. Atraumatic extraction of the hopeless tooth was performed. A CT Scan was taken immediately after extraction to quantify the ridge defect and also to plan the graft harvest from mandibular chin region (Fig. 1b,c,d)

Two vertical incisions were made at the line angles of both adjacent teeth. A full thickness flap was reflected extending 2mm beyond the base of the defect and the deficient ridge was thoroughly curetted and irrigated with saline (Fig. 2a). The irregularities of the recipient site were corrected conservatively with a round diamond bur. The defect was measured from deepest point of the deficient ridge from a fixed reference point i.e., the mid-point of the cement-enamel junction of adjacent mesial tooth. Periosteal releasing incision was given for mucoperiosteal flap mobilization to obtain tension-free primary closure.

-Graft harvestation: For surgical access to chin area,sulcular incision (21) was given & a full-thickness flap was reflected from that point down to the inferior border of symphysis. Care was taken to avoid degloving of the facio-inferior aspects &the lingual aspect of symphysis to prevent pseudoprognatism and reduction in lower lip height. Mentalis muscle attachment on the mental protuberance was left undisturbed to retain support for the chin profile (Fig. 2b).

The chin graft harvesting was done according to the new guidelines described by Pommer *et al.* in 2008 (14). The distance of the superior osteotomy cut to the tooth apices was kept at least 8 mm. The lower border was kept intact with the 5 mm safety distance from the mental foramen (14). A trephine bur [227B.204 bur, Komet®, Germany] with an internal diameter was selected depending on defect of the recipient site and an adequate size diameter of trephine bur was used to remove bone core of desired length (14) (Fig. 2b). A bone scraper [Safescraper twist, Meta®, Italy] was used to harvest particulate autograft from the same donor site. The donor site was packed by placing PRF and interrupted suturing was done with a black monofilament polyamide non absorbable suture [4-0 Ethilon®, USA].

-Graft placement and suturing: The bone core was adapted at the recipient site by minimum levelling of the recipient bed and by trimming the block conservatively to fit the site. Intra-marrow perforations were created in the graft and it was stabilized with a titanium screw of adequate diameter and length to prevent mobility. The top of the graft was placed at the highest available bone level of the both proximal teeth (Fig. 2c). The composite graft, mixture of autogenous graft particles with bioactive Calcium-Phosphosilicate graft [500-1000 microns, Novabone Morsels®, USA], was placed as a filler under the resorbable membrane [RCM 6 membrane®, ACE surgical, USA]. A black monofilament polyamide non absorbable suture [4-0 Ethilon®, USA] was used to adapt the flap margins by giving interrupted horizontal mattress interspersed with simple interrupted sutures.

-Post-operative management: In order to minimize the hematoma formation, incision line dehiscence and infection; the patient was given a chin pressure bandage for a minimum of 3 days. Immediate provisional restoration was delivered. The patient was asked to apply ice pack over the operated area intermittently for first 12 hours post-surgery and in case prolonged bleeding persisted, patients were asked to report to the hospital. Patients were advised to avoid chewing or putting any kind of load on the temporary crown.

Drug prescription included Tab. Ketorolac DT 10 mg [Ketorolac] twice daily for 5 days, tab. Augmentin 625 mg [500 mg amoxycillin and potassium clavulanate equivalent to 125 mg clavulanic acid] for 5 days and twice-daily rinses with 0.12% chlorhexidine gluconate for 2 weeks.

The patients were recalled immediately after 24 hours for post-operative observation, and irrigation of the recipient and donor site was performed. On subsequent recall visits, pressure bandage was removed on a 3rd day and suture removal was performed after 2 weeks of surgery. Regular follow up was done at 1, 3, and 6 months.

-Statistical analysis: The statistical analysis of all the clinical and radiographic values was performed using SPSS version 16.0 software. Wilcoxon signed rank test was used to compare pre and post changes in ridge volume. The mean values of clinical and radiographic measurements [Height & Width] immediately after extraction and 6 months after augmentation were used for analysis. For the entire test, *p* values ≤0.05 and <0.001 were considered statistically significant and highly significant respectively.

## Results

All the patients completed the stipulated follow up and there were no dropouts. One patient was excluded from the study as the graft did not integrate into the recipient site and was explanted. Hence, all the analysis was done on 10 patients only.

At baseline, the soft tissue mean vertical height was 10.1 ± 1.4 mm. Six months after ridge augmentation, the mean value was found to be 9.7 ± 1.5 mm with a mean difference of 0.40 [*P*= 0.04, statistically significant]. The mean soft tissue horizontal width at baseline and after 6 months of augmentation was found to be 8.40 ± 1.35 mm and 8.80 ± 1.32 mm respectively with a mean increase of 0.40mm [*P*= 0.04, statistically significant] ( Table 1).

**Table 1:**
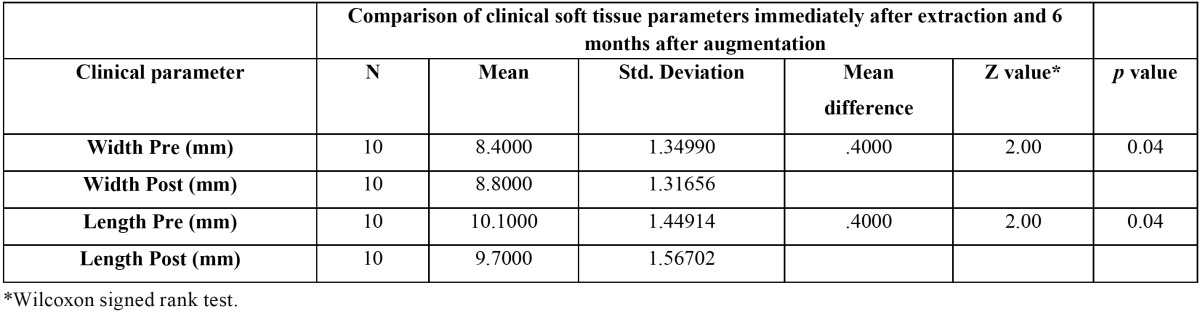
Comparison of clinical soft tissue parameters immediately after extraction and 6 months after augmentation.

At baseline, the mean defect depth from a fixed reference point [CEJ of adjacent tooth] measured on CT Scan was 11.9 ± 1.4 mm. Six months after ridge augmentation, the mean linear measurement was found to be 4.2 ± 1.1 mm with a mean reduction in the defect depth of 7.6 mm. The change in a defect depth after 6 months represented gain in the height and found to be statistically significant [*P*= 0.005]. The mean horizontal ridge width at the crest [from the highest point of the defect], as measured digitally from CT Scan, was 1.5 ± 0.4 mm. Six months after ridge augmentation, the mean value increased to 6.8 ± 2.6 mm. The mean difference in preoperative and post-operative horizontal ridge width was 5.2 mm which represents gain in the ridge width and was found to be statistically significant [*P*= 0.007] ( Table 2).

**Table 2:**
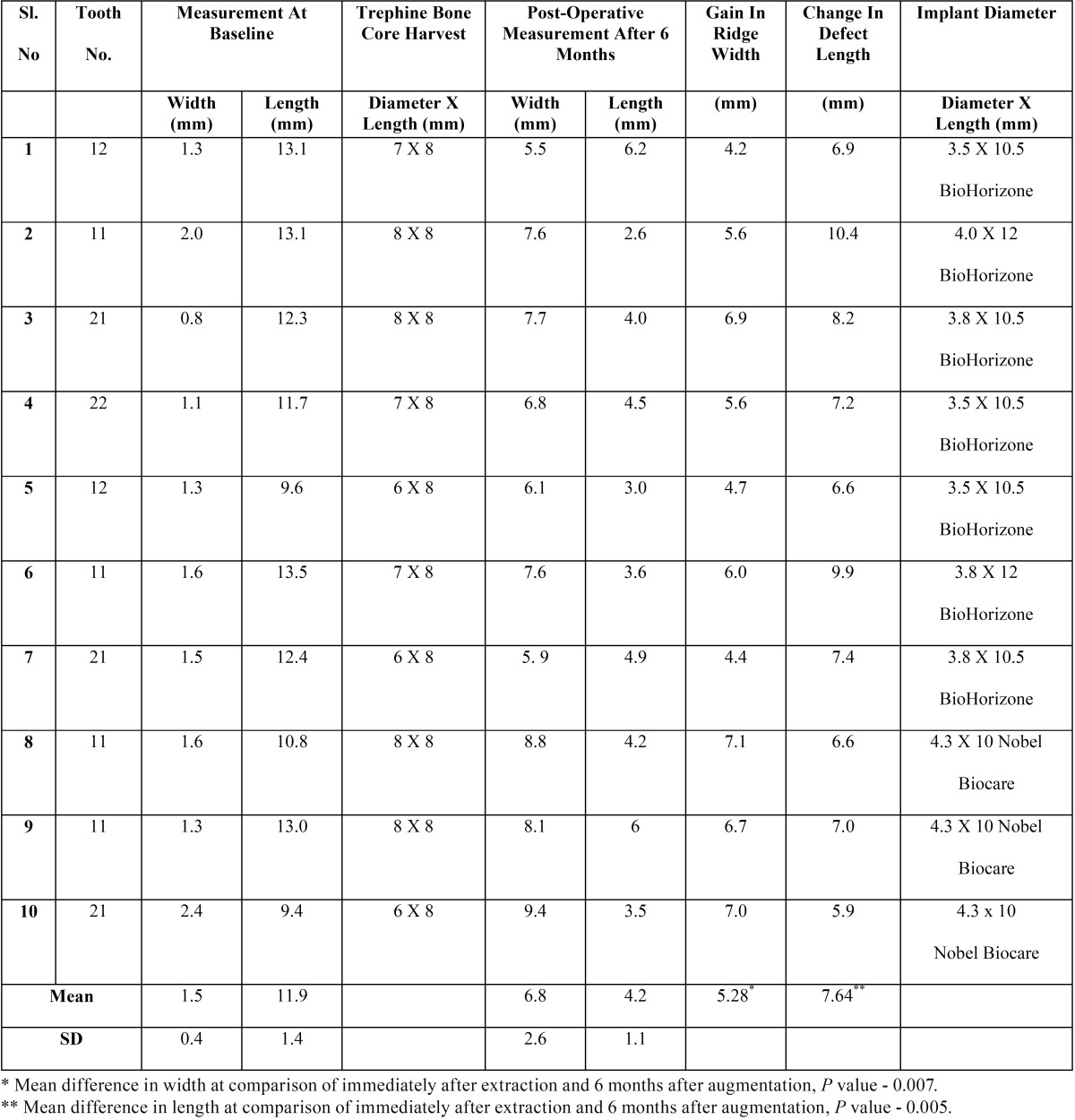
Baseline & 6 months post-operative radiographic measurements of the alveolar ridge (ct scan).

## Discussion

In the present scenario, the changing trend towards an evidence based dental treatment has led to the development of advanced techniques and treatment modalities. Over the past few decades implant dentistry has grown in scope due to the demonstrated success and predictability such that the present clinicians around the world consider it to be a form of standard care (4).

A systematic review by Tan *et al.* (22) on post extraction alveolar hard and soft tissue dimensional changes in humans, reported the horizontal bone loss [29-63%, 2.46-4.56 mm, at 6 months] was more substantial than vertical bone loss [11-22%, 0.8-1.5 mm, at 6 months] after tooth extraction. The greatest amount of bone loss was observed in the horizontal dimension involving mainly the facial aspect of the ridge. However in the presence of periodontal, periapical infections or vertical fracture with infected sinus tract, it may result in extensive bone resorption. When tooth extraction is mandated in such situations, it typically results in additional bone volume (10).

An excellent review on various surgical techniques for augmentation in the horizontally and vertically compromised alveolus has been given by Doonquah L *et al.* (23). Usually, after infected tooth extraction, patient has to wait 10-12 weeks for healing. In such cases, delayed ridge augmentation and implant procedures are mostly preferred which delays the overall treatment time. A periodontally hopeless tooth still is a taboo when considered in terms of IRA. However, here we have successfully reported a case series for predictable IRA at periodontally compromised extraction sites using an intramembranous autogenous chin block graft.

In the present case series, atraumatic extraction of tooth by periotome might have given additional benefit to prevent the trauma to the available thin buccal and palatal bony walls. Rationale behind thorough curettage and debridement of the defect was to eliminate all residual inflammatory tissue which if left behind, could expose the graft to the low pH environment of the socket, increase acid phosphatase activity, and compromise the bone regeneration process (24). Bleeding was induced at the apical end of the socket to increase the participation of endosteal bone-forming cells in the wound (25) and to avail the advantage of the regional acceleratory phenomenon (26).

Chin autograft, an intramembranous autogenous bone, has biological and immunological advantages in comparison to any allograft, xenografts, or alloplastic materials it contains viable cells [osteogenesis], BMP’s [osteoinduction]and a matrix for bone ingrowth [osteoconduction] (11,12). Other advantages are convenient surgical access, low morbidity, minimal donor site discomfort and the avoidance of cutaneous scars. It can be easily harvested in the office settings under local anaesthesia on an outpatient basis. Proximity of the donor and recipient sites reduce operative time and cost (12).

However, injury to MIC leading to neurosensory deficits of the lower lip, chin & anterior mandibular dentition is post-harvest concern. Safety rules as proposed by Misch have been shown to endanger the contents of MIC in 57% of the patients. New safety margins by Pommer *et al.* claims to reduce the risk of injury to MIC up to 16%. If proper patient selection is applied additionally, a residual risk of only 6% remains (14). As per the guideline, the symphysis can be used as a donor site in 56% of the patients to harvest a graft of 10mm diameter, in 74% of the patients for a graft of 8 mm diameter, and in 90% of the patients for a graft of 6 mm diameter. The residual 10% of the population are not suitable for chin bone harvesting (14). With the trephine technique, it is time saving, less tiresome procedure & easy to determine the required bone graft for augmentation of the extraction site.

In the course of the study, out of the 11 patients, one patient was excluded as the chin bone core did not integrate at the recipient site. The possible reason of the failure could be anxious-nervous nature of the patient and repeated tongue pressure to the grafted site [lower anterior] which led to inadequate primary stability of the block graft. Therefore, chin bone core was explanted 1 month post-operatively in relation to lower right central incisor area. Out of the remaining 10 patients, 2 patients [1 female and 1 male] showed incision line opening and membrane exposure by 2-3 mm after 15 days on suture removal. These patients were given Tab. Augmentin 625 mg twice daily for next 5 days and they were asked to continue 0.12% Chlorhexidine mouthrinse twice daily for upto 2 weeks as previously suggested (27). The area was allowed to heal by secondary intention and weekly follow-up was maintained. The healing of all other 8 patients was uneventful and no complications were reported. On recall visits, none of the patients reported complications related to recipient site. At the donor site, none of the patients reported neurosensory disturbance and lower anterior teeth were found to be vital on electric pulp testing.

In our study, mean loss of 0.4 mm in vertical height of soft tissue from baseline to 6 months after augmentation was observed. It was statistically significant [*P*<0.05]. However, in the absence of augmentation, Schropp *et al.* have demonstrated 0.9 mm of soft tissue collapse which is much greater (28). In essence; our immediate augmentation reduced the soft tissue collapse considerably but was not able to prevent it completely. When the baseline soft tissue width was compared with 6 months post-augmentation soft tissue width, a mean gain of 0.4 mm was observed. This emphasizes the importance of IRA. A previous study by Tan *et al.* demonstrated post-extraction gain of 0.4-0.5 mm of soft tissue width (22). In the present study, observed 0.4 mm of soft tissue gain in horizontal width was statistically significant.

On radiographic measurements [CT scan], the mean difference between the bone width at crest at baseline and 6-months post-augmentation was found to be 5.2 + 2.7 mm which was statistically significant [*P*= 0.007]. ( Table 2) Such a gain in bone width post-augmentation demonstrates the importance and effectiveness of IRA. These results are in agreement with previous study by Kfir *et al.* where a periodontally hopeless tooth was extracted and immediately recipient site was augmented using platelet rich fibrin and titanium mesh (10).

The mean difference in vertical defect depth as measured at baseline and 6 months post-augmentation [CT scan] was found to be 7.6 + 2.8 mm which was statistically significant [*P*= 0.005] ( Table 2). The reduction in defect depth actually indicates the amount of bone height gained. Thus a significant amount of vertical bone height was gained by IBA using autogenous chin bone graft. This is also in agreement with a previous study by Kfir *et al.* where a periodontally hopeless tooth was extracted and an implant site was developed using platelet rich fibrin and titanium mesh (10).

Thus in all the patients, the gain in ridge height and width was statistically significant [*P*<0.05] and sufficient to place an average of 3.5 - 4 mm diameter and 10 mm length implant fixture in the augmented site as a single or a staged approach ( Table 2, Fig. 3a,b). Seven patients received implants without any additional GBR and remaining 3 patients received vertical GBR simultaneously with implant placement using particulate graft and collagen membrane.

The consensus whether to perform immediate (29) or delayed GBR and implant placement (30) is still inconclusive. Kfir *et al.* found early membrane exposure in 7 out of 15 patients [47%] but no incidence of infection or early membrane removal was reported (10). Immediate augmentation after extraction of the periodontally hopeless tooth and delayed implant placement may be the preferred option, but this has not been supported by a randomized clinical trial (30). Difficulty in obtaining complete coverage of the extraction socket by soft tissue, early membrane exposure by epithelial dehiscence and infection of the augmented site remains concern for IRA surgery (10).

## Conclusions

Within the limitation of the present study, the preliminary data obtained indicates that the use of atraumatic extraction, IRA with autogenous chin block graft can be done to effectively prevent bone loss as well as soft tissue collapse immediately after extraction of a periodontally hopeless tooth. With proper case selection, it can be a reliable, safe and predictable approach to redevelop sufficient soft and hard tissue volume and also minimizes the overall treatment time for implant therapy. Further long term studies with a larger sample size and a randomised controlled design are required for institution of IRA as a regular treatment modality.

Harvesting a cortico-cancellous chin bone core with a trephine bur following new safety guidelines was found to be easy and with no post-operative neurosensory morbidity to the lower anteriors and mandibular symphysis region.
